# Economic argument for citrate haemofiltration

**DOI:** 10.1186/cc9546

**Published:** 2011-03-11

**Authors:** J Patterson, D Laba, M Blunt

**Affiliations:** 1 Queen Elizabeth Hospital, King's Lynn, UK

## Introduction

Regional citrate anticoagulation is associated with increased mean filter life and greater completion of scheduled filter life compared with heparin [1]. Studies report mean filter lifespans of 44 hours [2] and that 80% of patients reach 72 hours [3]. The potential cost saving from this reduced filter kit purchase is only realised if the treatment is stopped due to filter clotting and needs to be recommenced. In order to identify this we set out to evaluate the filter life and stopping reason for CVVHF treatment in general critically ill patients.

## Methods

One hundred sequential patients receiving CVVHF were identified. For each patient, the number of treatments, filter life and reason for stopping treatment were recorded. A subset of treatments in which stopping was due to filter clotting and therapy resumed was identified. These were then analysed to see how many filtration sets could be saved if the filter life was 44 hours [2]. Sensitivity analysis was performed based on a 50% change in filter life improvement.

## Results

A total of 304 filter sets were used in 100 patients (one to 14 per patient) - median duration 18.3 hours (IQR 8.5 to 38.3) (Table 1). Cost analysis demonstrated 75 filters could be saved if filter lives were prolonged to 44 hours, equivalent to €4.01/treatment-hour (€3.26 to €5.03).

**Table 1:** Treatments by stopping reason

Stopping reason	Access	Filter clot	Elective	End therapy	Miscellaneous	Total
Treatment resumed	Yes	Yes	Yes	No	Yes	
*n*	10	149	41	100	4	304
Duration (median) (hours)	13.4	16	38.0	22.8	11.2	18.3

## Conclusions

Prolonged filter life associated with citrate CVVHF leads to a potential saving of €4.01/treatment-hour. This information is of benefit when considering the business case for introducing citrate continuous venovenous haemofiltration.
